# User Engagement and Experiences With an Online Unsupervised Tai Chi Program for People With Knee Osteoarthritis: Mixed Methods Process Evaluation Nested in a Randomized Controlled Trial

**DOI:** 10.2196/82115

**Published:** 2025-11-14

**Authors:** Shiyi Julia Zhu, Rana S Hinman, Rachel K Nelligan, Peixuan Li, Anurika P De Silva, Jenny Harrison, Alexander J Kimp, Kim L Bennell

**Affiliations:** 1 Department of Physiotherapy Centre for Health, Exercise and Sports Medicine University of Melbourne Melbourne Australia; 2 Methods and Implementation Support for Clinical and Health (MISCH) Research Hub, Faculty of Medicine Dentistry and Health Sciences The University of Melbourne Melbourne Australia; 3 Rising Moon Tai Chi School Melbourne Australia

**Keywords:** digital health, online intervention, self-managed exercise, mind-body exercise, user engagement, user experiences, exercise adherence

## Abstract

**Background:**

Knee osteoarthritis is a major global health burden, and exercise is a core recommended treatment. Tai Chi is an evidence-based exercise shown to improve symptoms in people with knee osteoarthritis. However, traditional in-person delivery can limit accessibility. To address this, we developed a 12-week unsupervised online Tai Chi intervention and demonstrated its clinical effectiveness in a randomized controlled trial (RCT). This RCT compared the Tai Chi program plus educational information and an exercise adherence support app (intervention) with online education alone (control) for people with knee osteoarthritis. While the intervention improved pain and function, participants’ engagement and experiences with the online delivery format remain unclear. Understanding these perspectives is critical for improving future digital exercise interventions.

**Objective:**

This study aims to explore user engagement and experiences with an online unsupervised Tai Chi program among people with knee osteoarthritis.

**Methods:**

Quantitative and qualitative process measures were collected via self-report questionnaires from 89 participants who were randomized into the intervention arm of the RCT. User engagement was assessed using quantitative measures, including the number of days per week Tai Chi was undertaken (with adherence defined as ≥ 2 days per week), use of the My Exercise Messages App (The University of Melbourne), and scores from the Exercise Adherence Rating Scale Section B. User experience was assessed using quantitative measures of satisfaction, likelihood of recommending the program, and perceived credibility, usability, and acceptability. Qualitative content analysis of open-text responses was conducted to explore both positive and negative aspects of the program.

**Results:**

Sixty-four (72%) participants completed the process measures. Among those, the mean (SD) age was 62.5 (6.6) years, and 42/64 (66%) were females. The mean (SD) number of days Tai Chi was undertaken per week was 2.3 (1.1), with 54/74 (73%) classified as “adherent.” Participants reported high satisfaction (median 9, IQR 7-10), a strong likelihood of recommending it to others (median 9, IQR 8-10), and perceived it as credible, usable, and acceptable. Many participants described the program as engaging and well-delivered, reporting a positive experience overall and gaining improvements in their knee condition. However, some expressed concerns with aspects of the program delivery (eg, sessions were too long and slow), encountered learning and technological challenges, and a few were dissatisfied with their outcomes.

**Conclusions:**

Of participants who completed the process measures, most were highly engaged with the Tai Chi program and reported a positive experience, although some had a less favorable experience. This free online Tai Chi program has the potential to enhance patient access to guideline-recommended exercise for osteoarthritis.

**International Registered Report Identifier (IRRID):**

RR2-10.1016/j.ocarto.2024.100536

## Introduction

Knee osteoarthritis is a leading cause of disability, affecting 654 million people globally in 2020 and placing a substantial burden on health care systems [1]. Exercise is a core component of osteoarthritis management and is recommended in many clinical guidelines [2,3]. Among various exercise options, Tai Chi is strongly recommended [2,3], with evidence demonstrating its effectiveness in improving pain, physical function, and quality of life when compared to usual care [4]. However, Tai Chi is traditionally delivered in person in a group setting and may not be accessible to everyone due to geographical, financial, and logistical barriers.

In response to these challenges, digitally delivered interventions for osteoarthritis management are rapidly expanding as a means of improving access to evidence-based health care [5,6]. In recent times, we developed and evaluated the efficacy of a 12-week online unsupervised Tai Chi program [7], designed to increase access to guideline-recommended exercise for people with knee osteoarthritis. In a randomized controlled trial (RCT), we found that this intervention led to greater improvements in pain (mean difference –1.4 units, 95% CI –2.1 to –0.7; *P*<.001) and function (mean difference –5.6 units, 95% CI –9 to –2.3; *P*<.001) compared to online education control in 178 people with knee osteoarthritis at 12 weeks [8]. We also found that more people in the intervention group achieved the minimal clinically important difference in pain and function than in the control group [8]. While the clinical effectiveness of this Tai Chi program has been established, we are unsure about participants’ experiences using the program and any challenges they may have encountered. Understanding how participants engaged with and experienced the program is essential for refinement and informing the development of future online unsupervised exercise programs.

Therefore, the aim of this study was to explore user engagement (adherence) and experiences (satisfaction, recommendation likelihood, credibility, usability, acceptability, and positive and negative aspects) with an online unsupervised Tai Chi intervention among people with knee osteoarthritis.

## Methods

### Overview

This was a mixed methods process evaluation involving participants who were randomized into the intervention group of the RETREAT RCT (ACTRN12623000780651). The trial compared the “My Joint Tai Chi” intervention comprising an unsupervised online Tai Chi program plus educational information and an exercise adherence support app (“My Exercise Messages” app) with online education control for people with knee osteoarthritis [9]. It enrolled 178 Australian participants, with 89 randomized to the Tai Chi group. The enrollment period was from August 2023 to December 2024. The process evaluation included both quantitative and qualitative measures obtained via self-reported questionnaires.

### Participants

For the overarching RCT, people were recruited from the Australia-wide community via online advertisements and social media. The main inclusion criteria were: knee osteoarthritis diagnosis using the National Institute for Health and Care Excellence clinical osteoarthritis criteria [10]; history of knee pain ≥ 3 months; knee pain on most days in the past month; knee pain in the past week during walking ≥ 4 on an 11-point numerical rating scale (NRS), and; home internet connection and a device with access to the internet. The RCT protocol [9] outlines the detailed eligibility criteria. For this study, only participants randomized to the Tai Chi intervention were eligible to be included.

### Intervention: “My Joint Tai Chi”

A detailed description of the intervention (“My Joint Tai Chi”) and its development has been published [7,9]. The intervention was delivered via a website [11] with the intervention design features summarized in Multimedia Appendix 1. Briefly, “My Joint Tai Chi” was co-developed by Tai Chi instructors, people with osteoarthritis, physiotherapists, and osteoarthritis researchers. The main component of the intervention was a Tai Chi program delivered as a series of 12 different prerecorded 45-mintue videos (one video per week, with each one to be performed 3 times per week for 12 weeks) of a Tai Chi lesson led by an experienced Tai Chi instructor (JH). Each video consisted of a warm-up, a cool-down, and a modified 10-form Yang-style Tai Chi sequence, specifically adapted for individuals with hip and/or knee osteoarthritis and no/minimal prior Tai Chi experience. The program began with basic movements, gradually introducing new movements each week to progressively increase the level of difficulty. The website also included educational information about Tai Chi, living with knee osteoarthritis, treatment options, and the benefits of exercise for osteoarthritis, as well as a recommendation to download and use the “My Exercise Messages” mobile app [12] to facilitate adherence to the Tai Chi program.

### Process Evaluation Measures

Participant characteristics were extracted from baseline data collected for the RCT. Data regarding the process measures were collected via self-reported questionnaires administered 12 weeks after randomization in the trial, unless otherwise stated below. All data were collected via an online data collection platform REDCap [13,14].

The process evaluation measures collected were the following:

User engagement measures:

1. Number of days per week Tai Chi program undertaken: every 2 weeks post randomization (at 2,4,6,8,10, and 12 weeks), participants were asked, “In the past 2 weeks, on how many days did you do the ‘My Joint Tai Chi program’?” via email by the researcher (SZ). The average number of days per week Tai Chi was performed was calculated at each time point and across the entire 12-week period. The 12-week average was derived from data collected at each time point. If data were missing for certain weeks, those weeks were excluded from the calculation, and the average was based only on the weeks with available data. Participants were also classified as “adherent” if they reported doing Tai Chi ≥ 2 days per week, and all other participants were classified as “nonadherent.” From this, we reported the percentage of participants adherent or nonadherent at each fortnightly interval. We also recalculated this by including and categorizing participants who did not provide this data as nonadherent.
2. Exercise Adherence Rating Scale (EARS) section B [15]: participants were asked to answer 6 questions regarding their Tai Chi program adherence, each rated on a 5-point Likert scale from 0=“completely agree” to 4=“completely disagree”. Total score ranges from 0 to 24, with higher scores indicating better adherence.
3. Use of My Exercise Messages App: participants were asked, “Did you use the My Exercise Messages App at all in the past 3 months?” with response options of Yes or No.
4. Access to website: data obtained from website analytics showing the number of participants who accessed the website at least once.

User experience measures:

1. Overall satisfaction with Tai Chi program: participants were asked to score on an 11-point NRS for “Overall, how satisfied are you with the ‘My Joint Tai Chi’ program?” from 0=“not at all satisfied” to 10=“extremely satisfied.”
2. Likelihood of recommending Tai Chi program to others: participants were asked to score on an 11-point NRS for “How likely would you be to recommend the ‘My Joint Tai Chi’ program to others with knee osteoarthritis?” from 0=“not at all likely” to 10=“extremely likely.”
3. Credibility of Tai Chi program: participants were asked to score two items regarding how logical and trustworthy the “My Joint Tai Chi” program was, using an 11-point NRS from 0=“not at all” and 10=“extremely,” and one item regarding how believable the program was, using an 11-point NRS from 0=“not at all” and 10=“fully.” Higher scores indicate that the program is more logical, believable, or trustworthy.
4. Usability of Tai Chi program: participants were asked to score the usability of “My Joint Tai Chi” program using all 10 items from the System Usability Scale (SUS) [16], using a 5-point Likert scale from 1=“Strongly disagree” to 5=“Strongly agree.” The score was converted to a range from 0 to 100, with higher scores indicating the higher usability.
5. Acceptability of Tai Chi program: participants were asked to score the acceptability of the “My Joint Tai Chi” program using all 8 items from the Theoretical Framework of Acceptability (TFA) Questionnaire [17], each with 5 Likert response options from 1 to 5. Items 2, 3, and 7 were reverse scored. To generate a single acceptability score, the total mean score of the first 7 TFA items, or the single score for item 8 regarding general acceptability, were used. A higher score indicates higher acceptability.
6. Positive and negative aspects of the Tai Chi program: participants were asked to provide up to three open-text answers each to “List the most positive aspects” and “List the most negative aspects” of the program. Providing responses to these open-text questions was optional.

### Data Analysis

Quantitative measures were summarized as mean (SD) or median (IQR) for continuous variables, and counts or percentages for categorical variables.

For the open-text responses about the positive and negative aspects of the program, a qualitative inductive content analysis approach was used [18]. All open-text data were exported to Microsoft Excel and subsequently analyzed in NVIVO (QSR International Pty Ltd 2015, released 2024). Two researchers (SZ and JP) independently conducted the initial analysis, beginning by reading through all responses to familiarize themselves with the dataset. Date was deemed invalid and removed if the answer was not related to either positive or negative aspects of the program. The remaining data were then coded separately for each question by identifying “units of meaning” within participant responses that reflected either positive or negative aspects of the program. Researchers avoid using preconceived categories, instead immersing themselves in the data to allow new insights and thematic categories to emerge [19]. Each researcher independently generated preliminary themes and subthemes by organizing related codes and distinguishing them from unrelated ones. These themes and subthemes were labelled and defined, with a focus on manifest content [18,20]. Throughout the process, the researchers iteratively moved between the research question, original data, coding, and development of themes to ensure coherence and accuracy. The codes, subthemes, and themes were then discussed with the broader research team, initially with RN and subsequently with KB and RH, to consider alternative categorizations of the data (such as merging or reorganizing themes) and their descriptions.

### Ethical Considerations

Informed consent was obtained from participants before their completion of baseline questionnaires via online forms using REDCap (Research Electronic Data Capture) [13,14]. Ethics approval was obtained (Reference number 2023-26838-47588-6) in May 2023 from the Human Research Ethics Committee of the University of Melbourne. All data collected in this study were deidentified and stored on secure university servers, accessible only to the researchers using a password.

## Results

### Participant Characteristics

Of the 89 participants allocated to the Tai Chi group in the RCT, 64/89 (72%) completed all process measures at 12 weeks. Table 1 presents the baseline characteristics, and Multimedia Appendix 2 presents the primary outcomes of participants who did and did not complete the process measures. No major differences were identified between these subgroups. The number of participants who self-reported their Tai Chi sessions via fortnightly questionnaires was at its lowest (56/89, 63%) during weeks eight and ten, and at its highest (67/89, 75%) during week two. A total of 74 (83%) participants completed at least one questionnaire across the 12 weeks, while 15 (17%) did not complete any questionnaire at any time point.

**Table 1:** Baseline characteristics of Tai Chi group participants who provided all process measures at 12 weeks and those participants who did not.

Characteristic					Incomplete process measures (n=25)												Complete process measures (n=64)										
Knee pain during walking (NRS), mean (SD)					6.3 (1.2)												6.1 (1.2)										
Physical function (WOMAC), mean (SD)					29.3 (8.3)												28.2 (8.7)										
Age (years), mean (SD)					60.9 (8.9)												62.5 (6.6)										
**Sex, n (%)**																											
			Male				8 (32)												22 (34)								
			Female				17 (68)												42 (66)								
**Gender, n (%)**																											
			Man			8 (32)												22 (34)									
			Woman	16 (64)												41 (64)											
			Nonbinary	1 (4)												1 (2)											
Height (m), mean (SD)					1.7 (0.1)												1.7 (0.1)										
Body mass (kg), mean (SD)					91.9 (22.1)												86.2 (21.5)										
BMI (kg/m^2^), mean (SD)					32.6 (9.0)												30.4 (7.1)										
**Ethnicity, n (%)**																											
			Australian or New Zealander										17 (68)												50 (78)		
			Aboriginal and Torres Strait Islander										1 (4)												0 (0)		
			European										2 (8)												6 (9)		
			Asian										2 (8)												7 (11)		
			North African & Middle Eastern										1 (4)												0 (0)		
			Other										2 (8)												1 (2)		
**Education level, n (%)**																											
			Did not complete primary school										1 (4)												0 (0)		
			Secondary/high school										6 (24)												8 (12)		
			Trade or trade certificate										3 (12)												4 (6)		
			University or tertiary institute										10 (40)												42 (66)		
			Higher university degree										5 (20)												10 (16)		
			Currently in paid employment										15 (60)												39 (61)		
**Geographical location, n (%)**																											
	Major cities									18 (72)												36 (56)					
	Inner regional									6 (24)												21 (33)					
	Outer regional									1 (4)												5 (8)					
	Remote									0 (0)												2 (3)					
**Most painful knee**																											
	Right knee, n (%)										15 (60)												30 (47)				
	Left knee, n (%)										10 (40)												34 (53)				
		Knee symptom duration (years), median (IQR)		6 (4-20)												8 (2-46)											
**Problems in other joints, n (%)**																											
	Nonstudy knee							4 (16)												20 (31)							
	Upper body							9 (36)												21 (33)							
	Back and neck							9 (36)												20 (31)							
	Hip							6 (24)												14 (22)							
	Foot or ankle							10 (40)												10 (16)							
**Joint replacement, n (%)**																											
	Nonstudy knee						1 (4)												1 (2)								
	Hip						3 (12)												1 (2)								
**Comorbid conditions, n (%)**																											
	≥1 comorbid condition												23 (92)												57 (89)		
	Heart disease												0 (0)												6 (9)		
	High blood pressure												6 (24)												24 (38)		
	Lung disease												1 (4)												3 (5)		
	Diabetes												2 (8)												2 (3)		
	Ulcer or stomach disease												1 (4)												1 (2)		
	Kidney disease												0 (0)												1 (2)		
	Liver disease			0 (0)												0 (0)											
	Anemia or other blood disease			0 (0)												0 (0)											
	Cancer			0 (0)												3 (5)											
	Depression			3 (12)												7 (11)											
	Back pain			8 (32)												25 (39)											
	Rheumatoid arthritis			0 (0)												0 (0)											
	Other medical problems			7 (28)												15 (23)											
**Current oral medication use for knee pain^a^, n (%)**																											
	≥1 medication used													18 (72)												46 (72)	
	Analgesia or paracetamol combinations													16 (64)												40 (62)	
	Anti-inflammatory tablets or capsules													9 (36)												30 (47)	
	Oral corticosteroids													0 (0)												1 (2)	
	Oral opioids													0 (0)												0 (0)	
**Treatments for knee in last 3 months**																											
	≥1 treatment, n (%)								14 (56)												32 (50)						
	Massage or manual therapy, n (%)								11 (44)												23 (36)						
	Gait aid, n (%)								4 (16)												4 (6)						
	Electrotherapy^b^, n (%)								1 (4)												4 (6)						
	Joint injections, n (%)								3 (12)												2 (3)						
	Acupuncture, n (%)								4 (17)												4 (6)						
	Information or education course, n (%)								3 (12)												7 (11)						
	Herbal therapies, n (%)								1 (4)												13 (20)						
	Past experience with Tai Chi, n (%)														1 (4)												5 (8)
	Physical activity level (hours per week)^c^, median (IQR)														20 (9-32)												20 (13-28)
**Tai Chi expectations^d^, n (%)**																											
	Minimal improvement											0 (0)												6 (9)			
	Moderate improvement											17 (68)												34 (53)			
	Large improvement											8 (32)												23 (36)			
	Complete recovery											0 (0)												1 (2)			
**Health professional consultation^e^, n (%)**																											
	General practitioner							13 (52)												30 (47)							
	Physiotherapist							7 (28)												11 (17)							
	Exercise physiologist							1 (4)												3 (5)							
	Dietician								2 (8)												0 (0)						
	Psychologist								0 (0)												0 (0)						
	Pharmacist								5 (20)												15 (23)						
	Podiatrist								2 (8)												4 (6)						
	Occupational therapist								2 (8)												0 (0)						
	Rheumatologist								0 (0)												0 (0)						
	Sports and exercise physician								0 (0)												0 (0)						
	Orthopedic surgeon								2 (8)												1 (2)						
	Other								2 (8)												3 (5)						

^a^Defined as ≥ 1 time per week over the last 4 weeks.

^b^Electrotherapy includes transcutaneous electrical nerve stimulation, low-level laser, and ultrasound therapy.

^c^Measured using IPEQ-W (incidental and planned exercise questionnaire) with 10 questions regarding frequency and duration of incidental and planned walking, sport, and recreational activities over the past 7 days. Scores were calculated as the product of the frequency and duration scores to create a total duration for the week score, where higher scores indicate higher levels of activity.

^d^Scored from a question asking about expectation of study treatment outcomes, with scores on a 5-point Likert scale from “1=no effect at all” to “5=complete recovery.”

^e^Reported yes or no if any of the listed health professionals were visited for a knee condition in the past 3 months.

### User Engagement Measures

**Figure 1:**
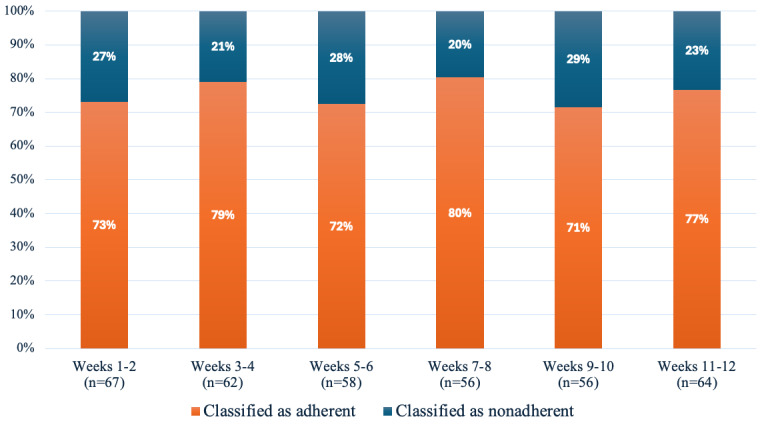
Fortnightly adherence rates in the Tai Chi group expressed as the percentage of participants classified as adherent (Tai Chi performed on average 2 or more days per week) and nonadherent.

Adherence to the Tai Chi program is shown in Table 2. Among those who provided adherence data (n=74), the average number of days Tai Chi was performed per week over weeks 1-12 was 2.3 (SD 1.1), with 54 (73%) participants achieving “acceptable” adherence (doing Tai Chi ≥ 2 days per week). The proportion of participants classified as adherent in each fortnightly period was stable throughout the 12 weeks, ranging from 71% in weeks 10 to 80% in weeks 8 (Figure 1). The adherence rate was lower when including those who did not provide adherence data and were categorized as nonadherence (Multimedia Appendix 3). The EARS also demonstrated high adherence to the program, with a median score of 20 (IQR 13–23.5) out of 24 reported at 12 weeks.

**Table 2:** Adherence measures in the Tai Chi group.

Adherence Measures				Tai Chi (n=89)		
**Average number of days Tai Chi performed per week^a^**						
	**Weeks, mean (SD)**					
		1-2			2.4 (1.1)	
		3-4			2.6 (1.3)	
		5-6			2.5 (1.3)	
		7-8			2.7 (1.3)	
		9-10			2.4 (1.3)	
		11-12			2.4 (1.3)	
	Average number of days Tai Chi performed per week over weeks 1-12^b^, mean (SD)		2.3 (1.1)			
	Classified adherent from data collected over week 1-12^b,c^, n/N (%)		54/74 (73)			
	Exercise Adherence Rating Scale Section B^d^, median (IQR)		20.0 (13.0-23.5)			
	Use of “My Exercise Messages” App^e^, n/N (%)		48/64 (75)			
	Access to website^f^, n/N (%)		86/89 (97)			

^a^Self-reported throughout questionnaire sent at 2, 4, 6, 8, 10, and 12 weeks; 67 answered at 2 weeks, 62 answered at 4 weeks, 58 answered at 6 weeks, 56 answered at 8 weeks and 10 weeks, 64 answered at 12 weeks.

^b^74 participants in Tai Chi group answered at least one questionnaire sent at 2, 4, 6, 8, 10, and 12 weeks.15 participants did not answer any questionnaire at any time point.

^c^Adherent defined as Tai Chi performed on average 2 or more days per week.

^d^Scores range from 0-24, higher scores indicating better adherence. Results obtained from 64 participants who answered this questionnaire.

^e^Participants were asked, “Did you use the My Exercises Message App at all in the past 3 months?” with response options of Yes/No. 64 participants answered this question.

^f^Data obtained from website analytics showing the number of participants who accessed the website at least once.

### User Experience Measures

Five responses were classified as invalid. Sixty-two participants provided at least one response to these questions, while 27 participants did not provide any response. Participants reported high satisfaction and a strong likelihood of recommending the program to others. Also, they perceived the program to be highly credible, acceptable, and usable (Table 3).

**Table 3:** User experiences (satisfaction, recommendation, credibility, usability, and acceptability) with the Tai Chi program.

User experience			Median (IQR)
Overall satisfaction with program^a^			9 (7-10)
Likelihood of recommending program^b^			9 (8-10)
**Credibility of program^c^**			
	1. How logical the program was	9 (7-10)	
	2. How believable the program was to help knee problem	6 (5-7)	
	3. How trustworthy the program was	9 (8-10)	
	Usability of program^d^	85 (72.5-95)	
**Acceptability of program^e^**			
	Average score from Questions 1-7	4 (3.6-4.3)	
	General acceptability score	5 (4-5)	

^a^Overall satisfaction with the program was scored on an 11-point numerical rating scale (NRS) from 0=“not at all satisfied” to 10=“extremely satisfied.” Results obtained from 66 participants who answered this question.

^b^Likelihood of recommending program was scored on an 11-point NRS from 0=“not at all likely” to 10=“extremely likely.” Results obtained from 65 participants who answered this question.

^c^Credibility of program was scored using 2 items regarding how logical and trustworthy the “My Joint Tai Chi” program was, using an 11-point NRS from 0=“not at all” and 10=“extremely,” and 1 item asking “Thinking back to when first starting the program, how believable the program was,” using an 11-point NRS from 0=“not at all” and 10=“fully.” Results obtained from 64 participants who answered these questions.

^d^Usability of program was scored using 10 items from System Usability Scale. Final score ranges from 0 to 100 with higher scores indicating higher usability. Results obtained from 64 participants who answered this questionnaire.

^e^Acceptability of program was scored using the Theoretical Framework of Acceptability (TFA) Questionnaire on a 5-point Likert scale with response options from 1 to 5. Items 2, 3, and 7 were reverse scored. To generate a single acceptability score, the total mean score of the first 7 TFA items, or the single score for item 8 regarding general acceptability were used. A higher score indicates higher acceptability. Results obtained from 64 participants who answered this questionnaire.

### Positive and Negative Aspects

Positive and negative aspects of the Tai Chi program are presented in Table 4, alongside themes, subthemes, and exemplar quotes.

Regarding positive aspects of the program, three themes were identified. First, participants described it as an engaging and well-delivered program. They appreciated the clear instructions and demonstrations from the instructor, and felt the program was well-structured and easy to understand. They also valued the comprehensive coverage of movements and information, delivered in a flexible format that allowed them to practice, rewatch, and rewind sessions without time or location constraints. Second, participants reported improvements in their physical and mental outcomes. For example, some noticed improvements in knee pain, function, body awareness, mental focus, and alertness. Third, participating in the Tai Chi program was considered a positive experience. Most participants found it relaxing and calming, and enjoyed the opportunity to learn something new.

In terms of the negative aspects, 4 themes were identified. First, participants raised concerns about program delivery. Some found the sessions too long and unrealistic in terms of the time required, while others felt the sessions were slow, boring, and repetitive. In contrast, a few people felt the sessions went too fast and wished they could be longer. Some participants also felt there was too much talking from the instructor. The prerecorded video format meant participants were unable to communicate with an instructor and gain feedback or check whether their movements were correct, which left some participants feeling frustrated. Second, participants reported various learning challenges. These included physical difficulty in performing complex movements, particularly due to a lack of coordination, and cognitive difficulty in remembering the movements. Third, technological challenges were identified. Issues such as poor internet connection and video presentation (eg, overly close-up shots) made learning more difficult for some. Fourth, some participants expressed dissatisfaction with their personal outcomes, feeling the program neither addressed their problems nor met their expectations.

In addition, suggestions for improvements were provided. Some participants wanted more information about Tai Chi before starting the program. There was also interest in having interactive components, such as an online forum for questions or follow-up opportunities with the instructor.

**Table 4:** Themes, subthemes, and exemplar quotes for positive and negative aspects of the Tai Chi program.

Themes and subthemes					Exemplar quotes
**Positive aspects of the program**					
	**Engaging and well-delivered program**				
		Clear instructions and demonstrations from the instructor		“Tai Chi teacher was very impressive. Explanations and demonstrations were clear and easy to follow. It was a joy to have someone so knowledgeable and professional.” “The instructor explained and demonstrated the movements very well.” “A very knowledgeable and patient instructor who was very encouraging.”	
		Well-structured and easy-to-understand		“Easy to understand and follow sequences.” “The 12 week program was really well paced.” “Well-presented and each lesson leads on from the previous.” “There was appropriate repetition to allow us to learn and understand.”	
		Good coverage of movements and information		“Good supply of information and content.” “The actual Tai Chi movements. Feeling the Tai Chi energy within.” “Good range of exercises.”	
		Flexible delivery mode		“Could do in your own home and at a time that suited me.” “I found that I could rewatch and rewind if I felt I needed more practice.” “Flexible timing.”	
	**Improved outcomes**				
		Improved physical well-being		“Most positive thing is that my knee does feel better.” “Found the movements helped my flexibility and strength.” “Increased my body awareness and mindful movement.” “I hope to start again and redo whole program as I have noticed huge improvement with my strength, stability and balance. But still have a long way to go.”	
		Improved mental well-being		“It improves my mental health like alertness.” “Meditation quality.” “Focus it provided.”	
	**A positive experience**				
		Relaxing and calming		“Enjoying learning Tai Chi warming and relaxation exercises.” “Calming, gentle and relaxing.” “I felt relaxed after completing the sessions.”	
		Good to learn something new		“Good to learn something new, hopefully perfecting the skills.” “Learning something new for life.” “Nice introduction to Tai Chi for a first timer.”	
**Negative aspects of the program**					
	**Concerns with program delivery**				
		Sessions were too long		“Extremely Boring. Too slow and monotonous.” “The slow pace at the beginning was difficult to adjust to.” “Too much repetition each session.”	
		**Disconfirming cases**			
			Too fast; Each session could go longer	“A bit too fast for me.” “Pace could be a little slower at the beginning of course.” “I sometimes wanted the session to go longer.”	
		Too much talking from the instructor		“Instructor spent a lot of time explaining things whilst we were held in one position.” “I felt the instructor spoke to us like children explaining the easiest movements in too much detail and repetitively as if we are totally stupid.” “Stopping mid exercise to explain a certain move.”	
		Lack of feedback		“Not being able to have two way communication.” “Not sure I am doing the right movement or steps on the skills.” “Lack of feedback, would be good to do in-person class in the first session.”	
	**Learning challenges**				
		Physical difficulty in learning complex movements, especially due to a lack of coordination		“Tai chi isn't easy for me.. body feet arm coordination challenged me.” “Some movements to me were difficult to grasp initially” “Unable to complete all tasks as easy as the instructor.”	
		Cognitive difficulty in remembering movements		“Having to remember most of the movements can be a bit daunting.” “I had great difficulty in remembering the moves, which I found frustrating and demotivating.”	
	**Technological challenges**				
		Video presentation		“Felt close-up views of exercises frustrating.” “It was very distracting zooming in on her feet. It was much easier to follow when zoomed out and watching her fully “It can be a bit confusing facing the screen and deciding which way is left or right.”	
		Poor internet connection		“Living remote, streaming sometimes difficult.” “Connectivity on my own system.”	
	**Dissatisfied with outcomes**				
		—^a^		“My shoulder issue was exacerbated a little with some movements so I modified it.” “Did not help me with my problem.” “(The program) could have been a bit more demanding for increasing knee strength and flexibility.”	

^a^Not applicable.

## Discussion

### Principal Findings

This process evaluation of a 12-week online unsupervised Tai Chi intervention showed that among participants who completed the measures, most were adherent to the Tai Chi program, reported high satisfaction with the program, a strong likelihood of recommending it to others, and perceived it as credible, usable, and acceptable. Many participants described the program as engaging and well delivered, reporting a positive experience and gaining improvements in their knee condition. However, some participants expressed concerns about aspects of the program delivery and encountered learning and technological challenges, with a few dissatisfied with their outcomes. To our knowledge, this is the first study to evaluate user engagement and experience with an online unsupervised Tai Chi intervention in people with knee osteoarthritis.

Overall engagement with the Tai Chi program was good, with nearly three quarters of participants deemed as having “acceptable” adherence. Compared to 2 other online unsupervised exercise programs we previously developed for knee osteoarthritis, one a 12-week yoga program [21] and the other a 6-month strengthening program [22], the EARS score in our study (median score of 20 out of a maximum score of 24) was higher than those reported in the other programs (both around 15-16) [21,22], suggesting that participants were more adherent with the Tai Chi than with these other programs. Furthermore, in contrast to another 9-week online unsupervised physical activity intervention for knee osteoarthritis (“Joint2move”) [23], which had an adherence rate of 46% (defined as 6 out of 9 modules completed) [24] and where module completion declined from 80% to 40% over time [23], adherence to our Tai Chi intervention remained stable (71%-80%) throughout the 12-week period, indicating sustained and consistent engagement. However, our adherence rates may be inflated as participants with missing adherence data were excluded from the calculation, which can introduce bias. Our recalculated adherence rates were lower when we included these participants and categorized them as nonadherent. One possible reason for the good engagement with our Tai Chi intervention is the incorporation of an adherence support tool, the “My Exercise Messages” app. This app was adapted from our previously developed, behavioral change theory-informed, automated SMS text messaging program, which showed improved self-reported adherence to unsupervised home exercise for people with knee osteoarthritis [25]. Given that 75% of our program completers reported using the app, it is possible that it contributed to the good adherence to our Tai Chi intervention. This is consistent with findings from systematic reviews showing that the use of behavior change techniques is associated with improved exercise adherence [26,27].

The generally positive user experiences with our Tai Chi program, as captured by the quantitative measures, align with findings from a systematic review [28] and a scoping review [29], showing that older adults reported high satisfaction, usability, and acceptability toward Tai Chi delivered remotely via synchronous videoconferencing. Specifically, satisfaction with the Tai Chi intervention (median of 9 out of 10) was slightly higher than that reported in our yoga program (mean of 8 out of 10) [21], and usability of the Tai Chi intervention (median of 85 out of 100) was higher than that of our strengthening program (mean of 5.3 out of 7) [22]. Under the credibility domain, the relatively low believability score (median of 6 out of 10) when participants first commenced the program likely reflects their initial uncertainty about Tai Chi, with their subsequent engagement and generally positive experiences suggesting that believability may have increased over time had this been remeasured. However, qualitative open-text responses in our study showed that while some participants described the program as well-structured with clear instructions, others found it too slow, repetitive, and boring due to excessive instructions. These contrasting experiences are consistent with our qualitative interview exploring in-depth experiences of a subgroup of 20 people who participated in this Tai Chi program [30], as well as other studies exploring home-based Tai Chi (delivered either as an addition to in-person classes or via videoconferencing) for people with chronic diseases (eg, lower back pain, dementia, and cystic fibrosis) where users also reported variable experiences [31-33]. Given that Tai Chi and yoga share similar elements of movement-based mind-body connection, we can draw further insight from 2 qualitative studies exploring yoga instructors’ experience of adapting in-person yoga classes to synchronized online delivery via videoconferencing [34,35]. These studies found that the online classes were intentionally designed to be slower, simpler, and more structured than face-to-face classes, where the instructor increased instructions and emphasized interoception (a technique of focusing on internal bodily sensations to improve mind-body awareness) [34-36]. As our Tai Chi program was delivered via pre-recorded videos without any instructor interaction with participants, we deliberately designed it to be slower and more repetitive to accommodate for the absence of hands-on assistance and direct feedback from an instructor. However, this teaching format might be perceived differently for individuals with variable learning preferences and needs.

The learning and technological challenges encountered by some participants may have also negatively impacted their experience with the program. Similar difficulties have been reinforced in our qualitative study [30] and reported in other studies of online exercise programs for older adults, where participants struggled to learn movements through a two-dimensional interface and experienced various internet-related issues [34,37-39]. The nature of an online unsupervised learning format may further reduce participants’ engagement, given the lack of face-to-face interaction and social support from being part of a group-based class. Collectively, our findings align with previous research showing that factors such as preferred teaching styles in mind-body exercise, movement complexity, digital literacy, and supervision are important in influencing participants’ engagement and experience with online mind-body exercise programs [29,40-42].

The fact that many people with knee osteoarthritis reported positive engagement and experiences with our online unsupervised Tai Chi program, but some others had less favorable experiences, indicates that there is no one-size-fits-all approach to exercise programs. This is supported by a Cochrane review showing similar symptomatic benefits can be obtained from a range of different exercise types, dosage, and delivery modes in knee osteoarthritis [43]. Instead, offering and improving access to a variety of exercise options that align with patient preferences may be important for optimizing engagement, adherence, and outcomes. For future implementation, providing additional information about the Tai Chi exercises (such as readings or videos) prior to program commencement may help participants better prepare for online unsupervised exercise programs.

In addition, offering a suite of optional shorter, prerecorded videos may enable users to select sessions that best suit their needs, while also helping to ensure they are adequately challenged. Finally, a hybrid approach that combines in-person sessions with digital Tai Chi could provide participants with initial instruction and feedback, enhancing their confidence before transitioning to independent online practice.

To date, more than 47,747 users from over 141 countries have accessed our freely-available unsupervised online-strengthening [44] and yoga [45] programs, highlighting the global demand for unsupervised home-based resources for osteoarthritis management. Our online Tai Chi program now adds to the growing suite of online exercise resources, providing an additional accessible option to support patient uptake of osteoarthritis exercises and potentially alleviate demands on health care services.

### Strengths and Limitations

Strengths of this study include a mixed methods process evaluation embedded within a robust RCT. By combining both quantitative and qualitative data, the study provided a more nuanced understanding of user engagement, program experiences, and considerations for future implementation. However, several limitations should be acknowledged. First, the process evaluation completion rate was moderate, with almost one-third of participants in the Tai Chi group not completing the evaluation. This may have introduced bias. For example, if noncompleters were those who either did not engage with the program or had less positive experience, the positive outcomes reported may have been overestimated, limiting the representativeness of the findings. Second, we were unable to capture accurate website continuous adherence metrics for the Tai Chi program, which would have provided a more objective measure of adherence. Instead, adherence data in this study were self-reported by participants, which may have introduced social desirability and recall biases that can affect the data accuracy [46,47]. However, a systematic review showed that self-reported diaries measuring the frequency of unsupervised interventions are the most commonly used method for assessing adherence [48]. While this approach has limitations, it remains a practical option when objective methods are not feasible. Future work could incorporate objective adherence metrics, such as those obtained from wearable technology or app-based logs to triangulate findings. Third, qualitative data were collected via an optional open text box allowing a maximum of 3 responses, which may mean our qualitative data may lack granularity. However, our qualitative interviews, published elsewhere [30], provide deeper information from a smaller subset of participants that complement the findings of the present study. Finally, participants were largely older adults, highly educated (university degree or higher), and located in major cities, and they were also required to own a device and access the internet. This may limit the generalizability of our findings to more culturally and linguistically diverse groups, socioeconomically disadvantaged populations, and people with lower digital or health literacy. Future studies could evaluate the program in broader populations to ensure its acceptability and applicability.

### Conclusion

Among participants who undertook a 12-week online unsupervised Tai Chi intervention and completed process measures, the majority were adherent with the weekly Tai Chi program, reported high satisfaction with the program and were likely to recommend it to others. The program was perceived as credible, usable, and acceptable. However, some participants expressed concerns related to program delivery, encountered learning and technological challenges, and a few were dissatisfied with their outcomes. This free and scalable Tai Chi intervention has the potential to enhance patient access to guideline-recommended osteoarthritis exercise.

## Appendix

Multimedia Appendix 1 Description of the online “My Joint Tai Chi” intervention (https://myjoint-taichi.org/).

Multimedia Appendix 2 Summary of the primary clinical trial outcomes of Tai Chi group participants who provided all process measures at 12 weeks and those participants who did not.

Multimedia Appendix 3 Fortnightly adherence rates in the Tai Chi group expressed as the percentage of participants classified as adherent (Tai Chi performed on average two or more days per week) and non-adherent, including those who did not provide adherence data categorized as non-adherent.

## Abbreviations

EARS: Exercise Adherence Rating Scale
NRS: numeric rating scales
RCT: randomized controlled trial
REDCap: Research Electronic Data Capture
SUS: System Usability Scale
TFA: Theoretical Framework of Acceptability

## Data Availability

The datasets generated or analyzed during this study are available from the corresponding author on reasonable request.
