# Supplementary material for: User Engagement and Experiences With an Online Unsupervised Tai Chi Program for People With Knee Osteoarthritis: Mixed Methods Process Evaluation Nested in a Randomized Controlled Trial
Source: JMIR Rehabil Assist Technol. 2025 Nov 14;12:e82115. doi: 10.2196/82115 (PMC12663698; doi:10.2196/82115)
Supplement: Multimedia Appendix 1 [file rehab_v12i1e82115_app1.docx]

## **Supplementary Online Content**

## **User engagement and experiences with an online unsupervised Tai Chi program for people with knee osteoarthritis: a mixed-method process evaluation nested in a randomised controlled trial.**

**Table 1. Description of the online “My Joint Tai Chi” intervention** (<https://myjoint-taichi.org/>)

The following information describes the four sections of the website describing the “My Joint Tai Chi” intervention:

1). 12-week unsupervised online Tai Chi program (“My Joint Tai Chi” program)

2). Information about Tai Chi

3). Information about living with knee osteoarthritis (OA), treatment options, the benefits of exercise for OA, the recommendation to do exercises and video interviews of OA experts (people with knee OA & OA researchers)

4). Information about the “My Exercise Messages” app to facilitate adherence to the Tai Chi program

| **A 12-week unsupervised online Tai Chi program (“My Joint Tai Chi” program)** | |
| --- | --- |
| This section contains an unsupervised online 12-week progressive Tai Chi program (“My Joint Tai Chi” program) guided by an instructor delivering the program in a series of 12 pre-recorded videos (each 40-45 mins, 1 per week). The central portion of each video uses a modified 10-form Yang style Tai Chi which involves slow and controlled movements. The sequence of the 10 movements is provided in the diagram below.  This section also provides an introduction to the Tai Chi program, how to prepare, information on managing exercise pain, optional resources including a Tai Chi skill video, a printable Tai Chi movement sequence (pdf), and suggestions for people about what to do after completing the 12-week program. | |
| **Diagram of the modified Yang style 10-form sequence in the program** | |
| **Form name** | **Diagram** |
| **1. Commencement** | 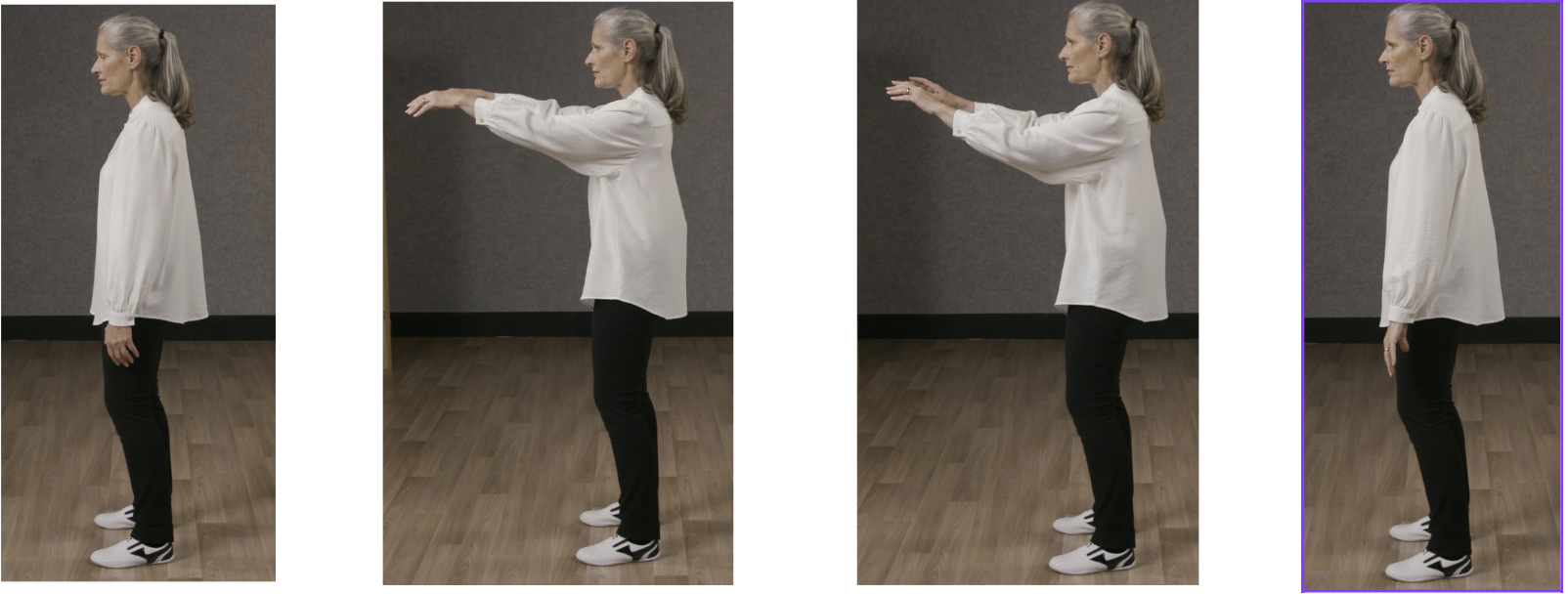 |
| **2. Repulse Monkey** | 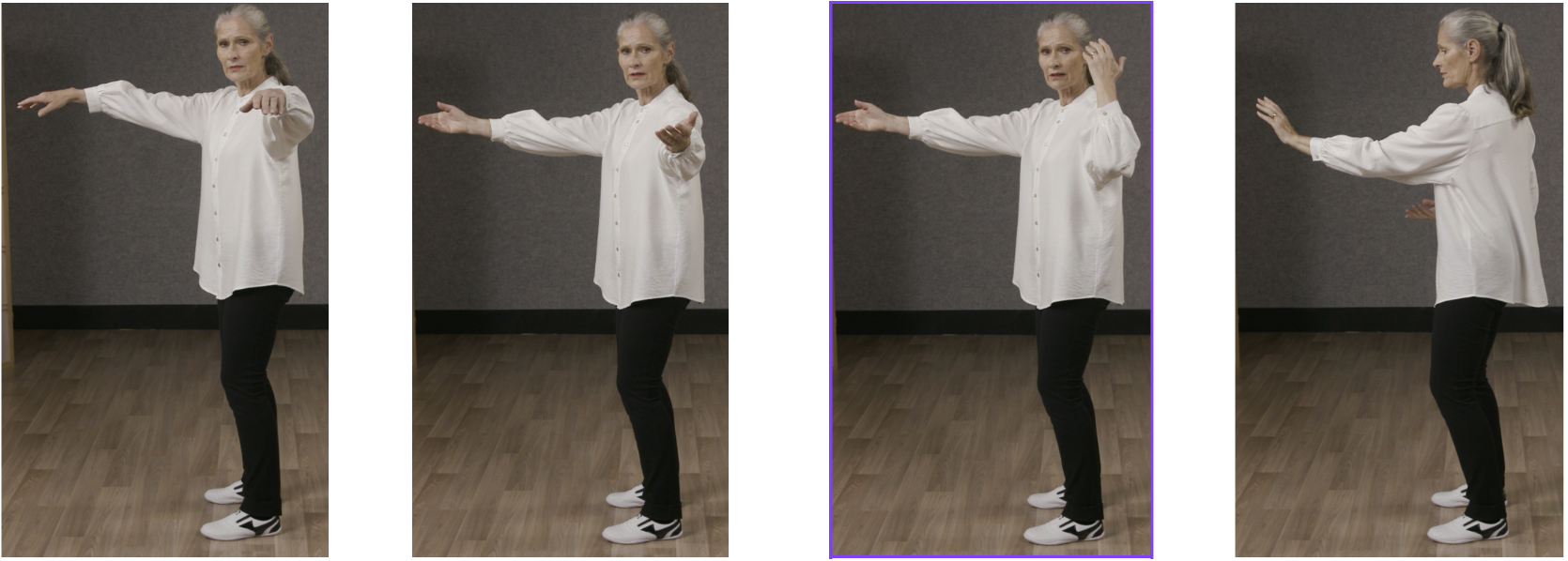 |
| **3. Brush Knee** | 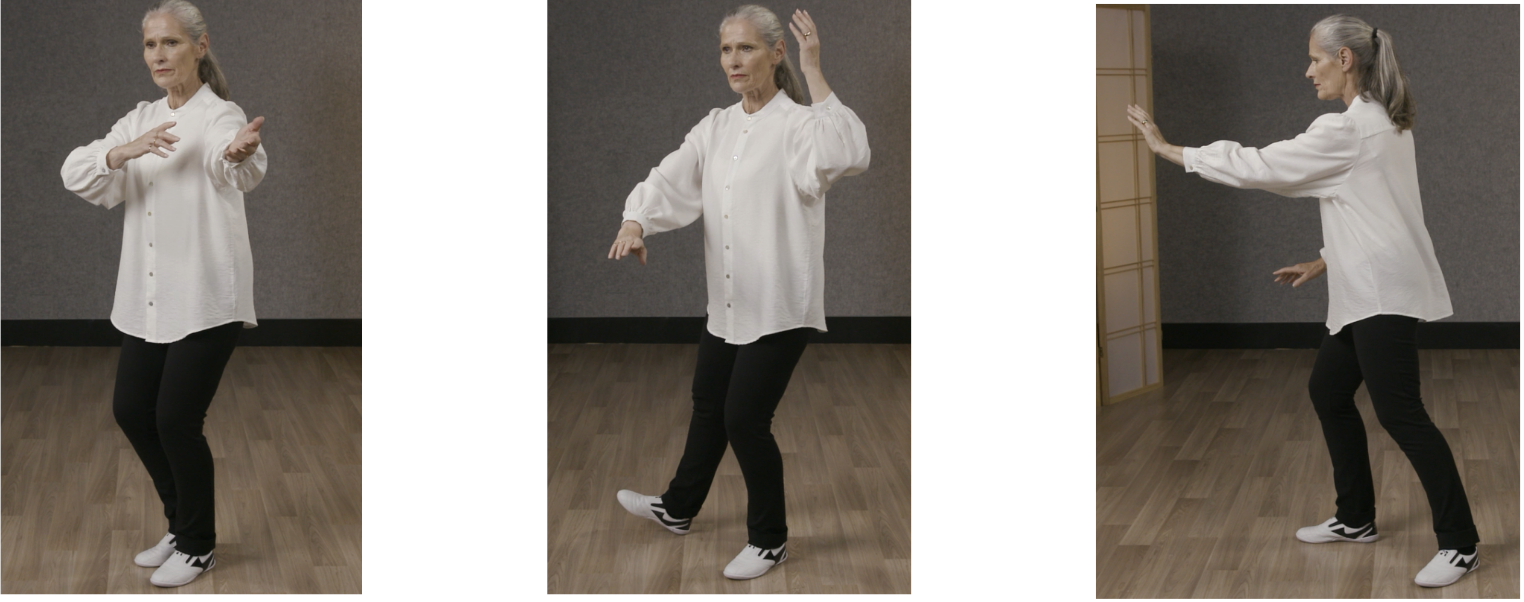 |
| **4. Part the Wild Horse’s Mane** | 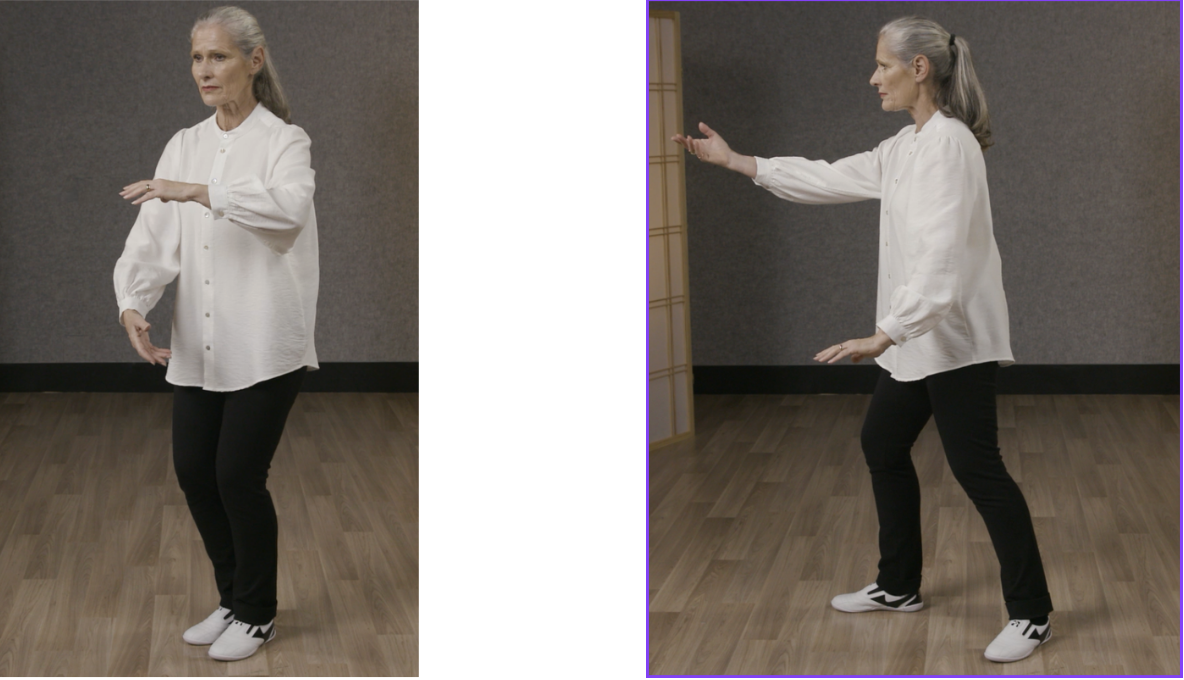 |
| **5. Cloud Hands** | 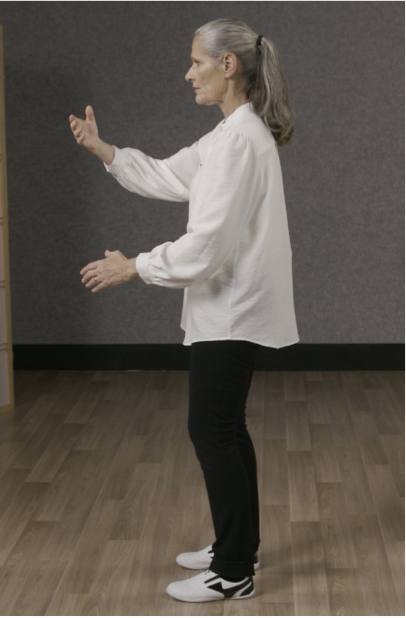 |
| **6. Golden Rooster Stands on One Leg** | 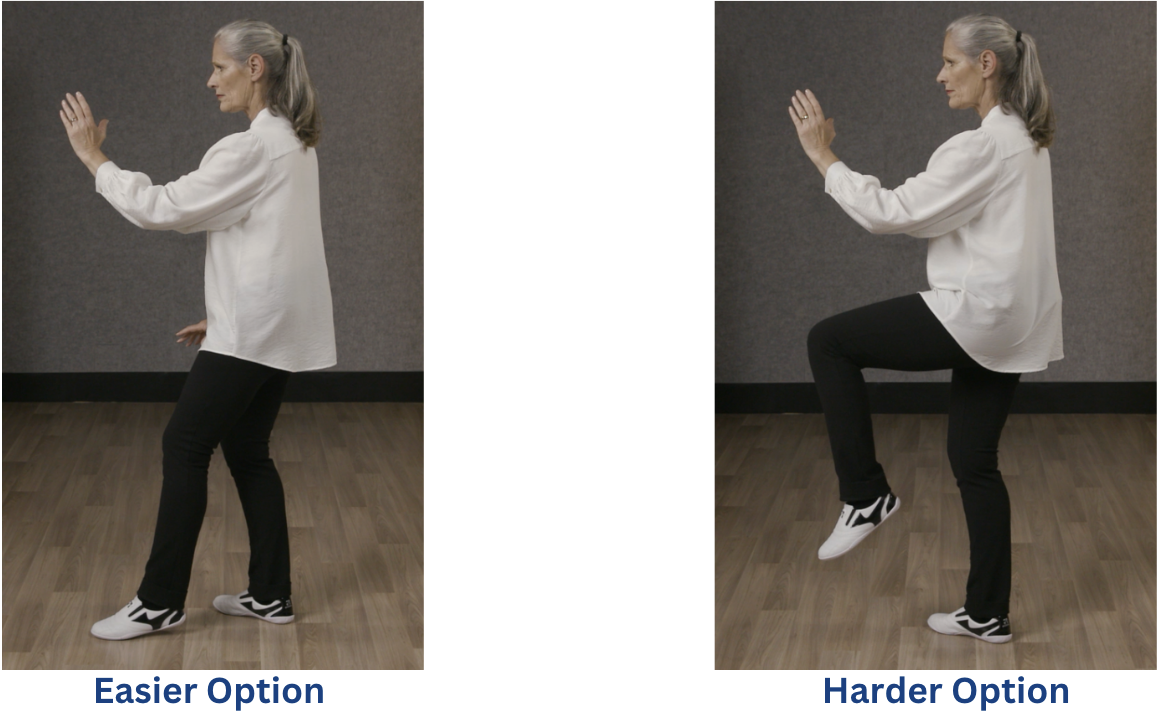 |
| **7. Kick with Heel** | 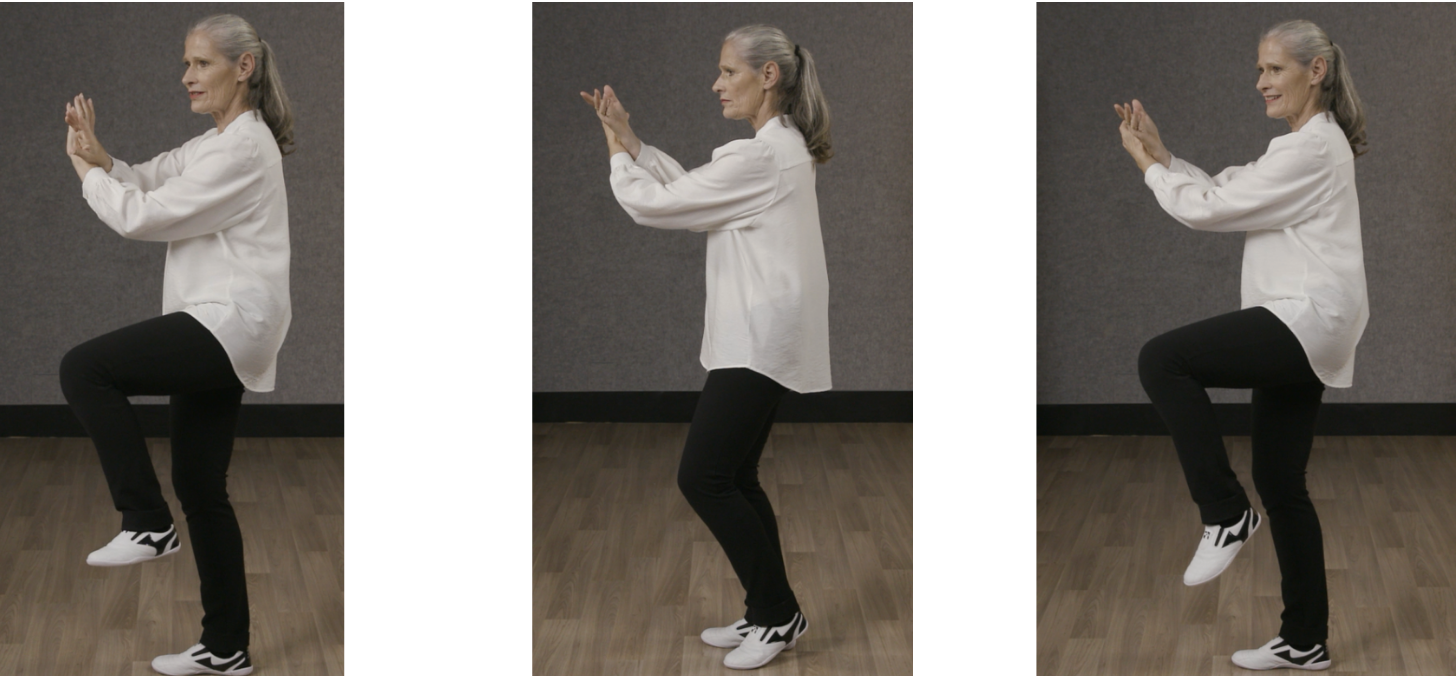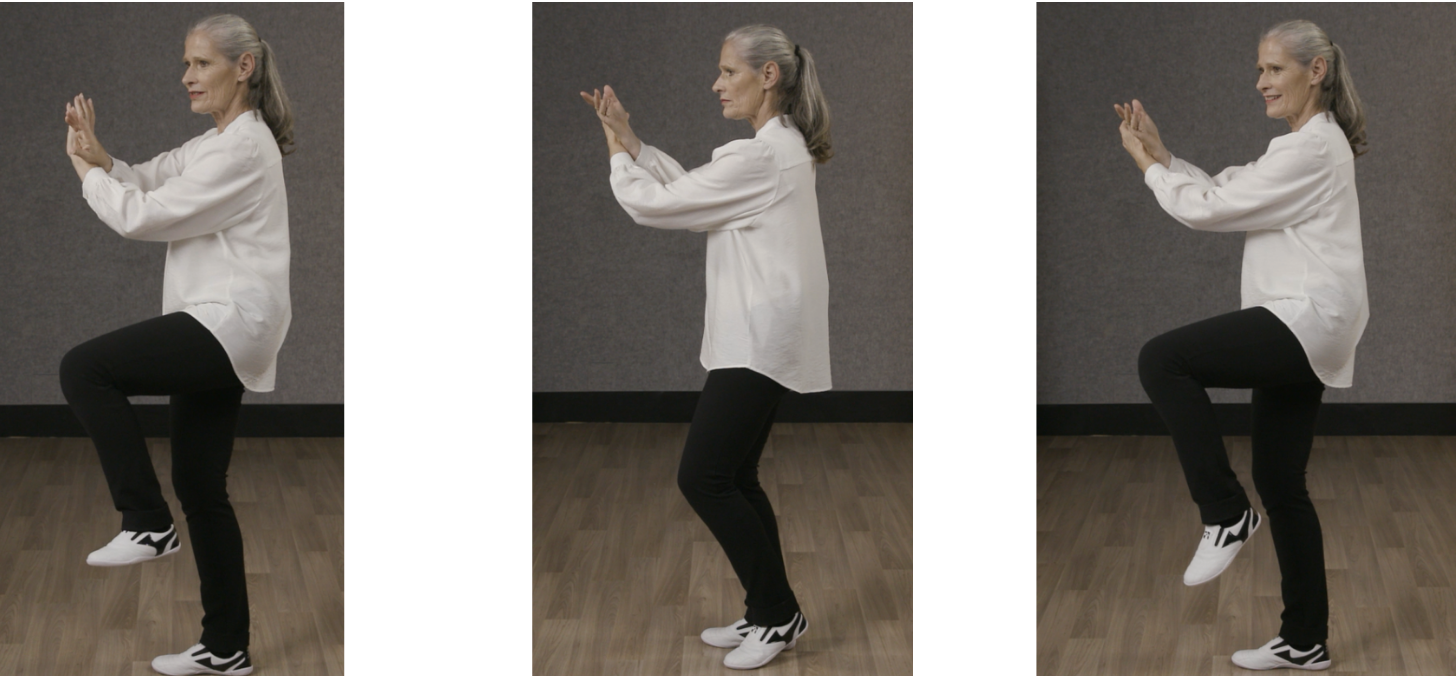 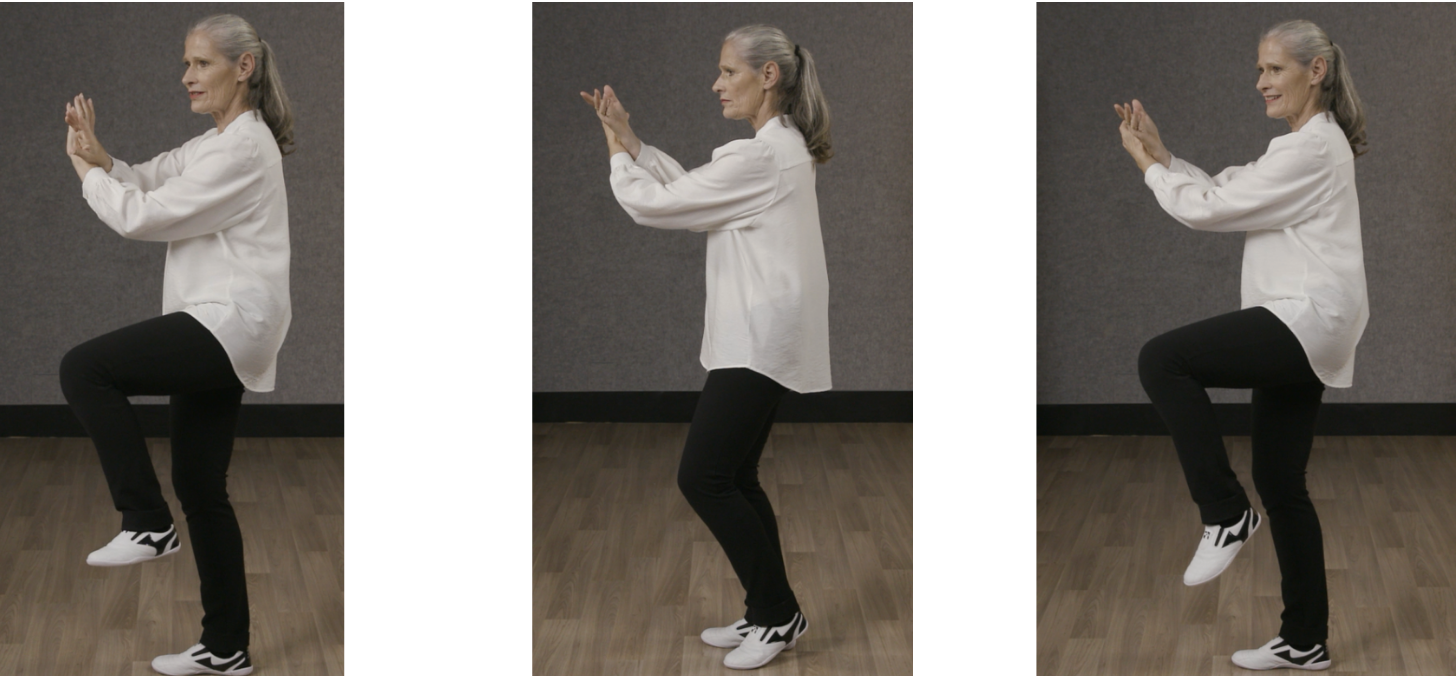  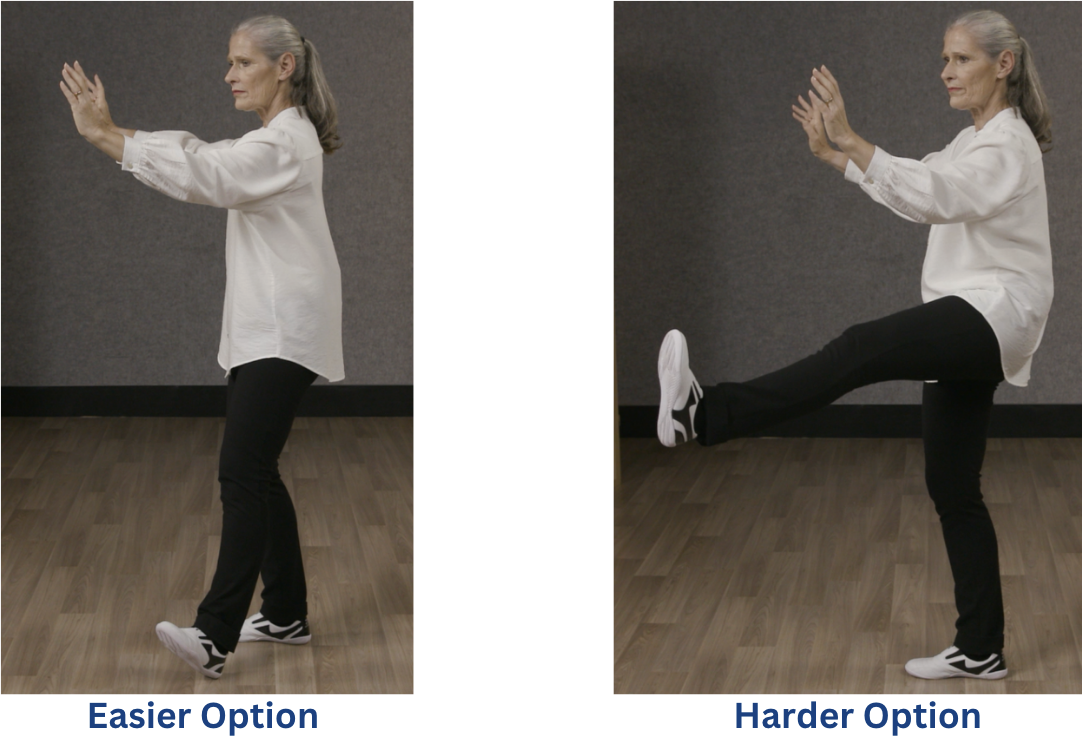 |
| **8. Stroke Peacock’s Tail/ Grasp Bird's Tail** | 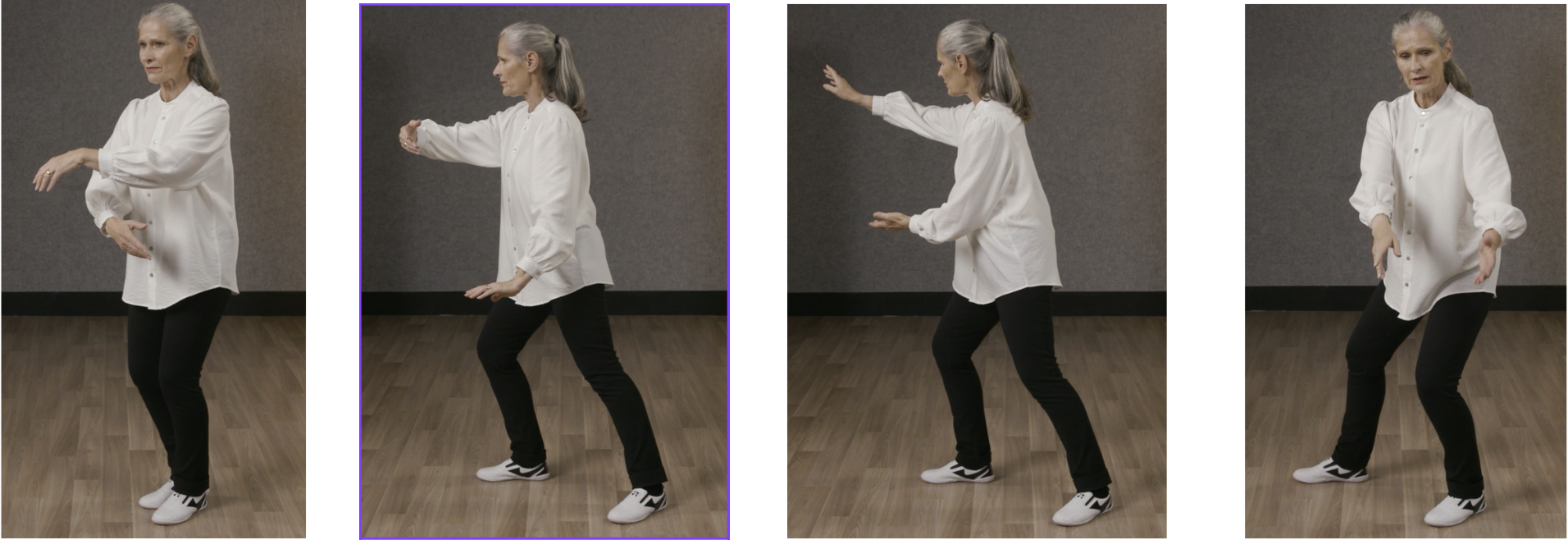  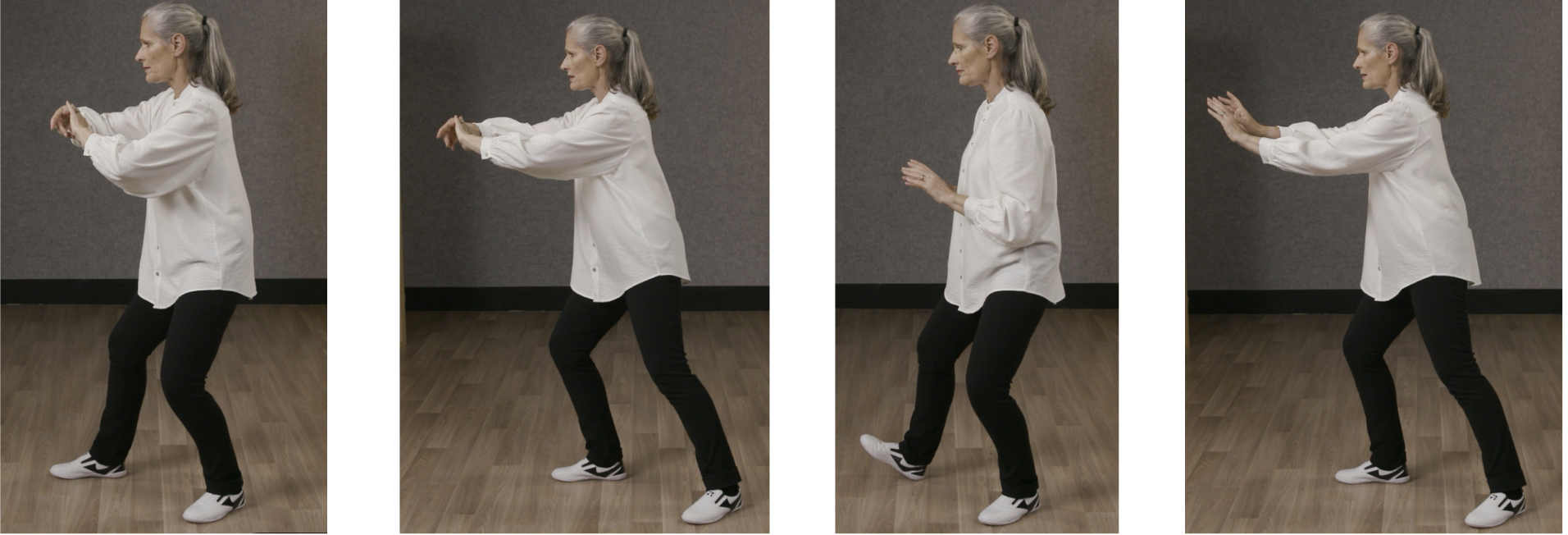 |
| **9. Embrace the Tiger** | 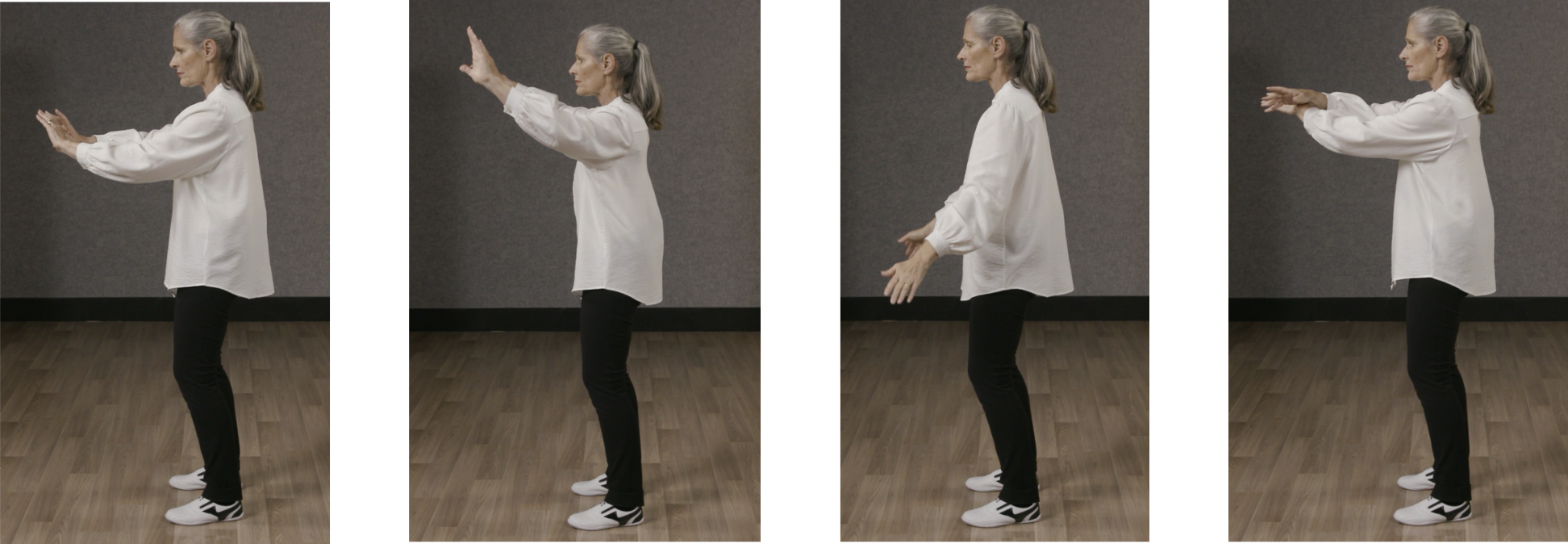 |
| **10. Closing** | 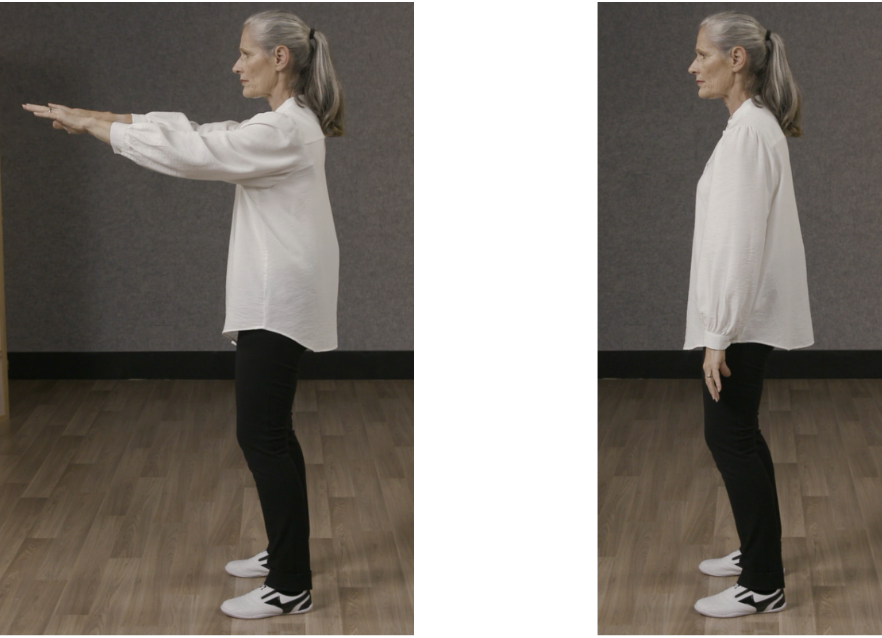 |
| **Information about Tai Chi** | |
| This section provides introductory information, including a definition of Tai Chi. It explains important principles of movement and outlines the health benefits associated with Tai Chi practice. | |
| **Information about OA and video interviews of OA experts (people with knee OA & OA researchers)** | |
| This section contains general information about living with knee OA, knee OA treatments, the benefits of exercise for OA and the recommendation to do exercises in a written format. Written information is complemented by video interviews of OA experts. The website recommends that participants read the educational material and watch the videos while doing the “My Joint Tai Chi program”. | |
| **Information about the “My Exercise Messages” app to facilitate Tai Chi adherence** | |
| The “My Exercises Messages” app was specifically designed for people with hip and/or knee OA to help them adhere to any exercise program. The “My Joint Tai Chi” intervention recommends users download and commence using the app in week 1 of their Tai Chi program and encourages them to use it for the full duration of the Tai Chi program (12 weeks). The app is free to download from the App Store (Apple devices) and Google Play Store (Android devices). After downloading the app, the app will prompt users to input i) their name; ii) the duration of the exercise program (12 weeks); and iii) the number of days per week they are aiming to complete their exercise (3 days per week). Seven days after setting up the app (and each week thereafter), they will receive a smartphone notification, from the app, prompting them to enter the app and record the number of days they completed their recommended Tai Chi exercise, in the past week. If they report less than 3 days in the past week, they will then be prompted to select a reason from a pre-specified list of common exercise barriers for people with OA. Users will then receive a tailored message containing a behaviour change technique suggestion to help them overcome their identified exercise barrier in the coming week. Users also receive two notifications each week containing messages designed to motivate regular exercise participation. | |
