# Supplementary material for: User Engagement and Experiences With an Online Unsupervised Tai Chi Program for People With Knee Osteoarthritis: Mixed Methods Process Evaluation Nested in a Randomized Controlled Trial
Source: JMIR Rehabil Assist Technol. 2025 Nov 14;12:e82115. doi: 10.2196/82115 (PMC12663698; doi:10.2196/82115)
Supplement: Multimedia Appendix 2 [file rehab_v12i1e82115_app2.docx]

**Multimedia Appendix 2**

**Summary of the primary clinical trial outcomes of Tai Chi group participants who provided all process measures at 12 weeks and those participants who did not.**

|  | **Incomplete process measures** | **Complete process measures** |
| --- | --- | --- |
|  | **N=25 ^a^** | **N=64** |
| Knee pain during walking (NRS) at baseline | 6.3 (1.2) | 6.1 (1.2) |
| Knee pain during walking (NRS) at 12 weeks | 3.9 (2.3) | 3.3 (2.2) |
| Change in Knee pain during walking (NRS)* | -2.4 (2.2) | -2.7 (2.2) |
| Physical function (WOMAC) at baseline | 29.3 (8.3) | 28.2 (8.7) |
| Physical function (WOMAC) at 12 weeks | 21.3 (13.1) | 15.3 (9.4) |
| Change in Physical function (WOMAC)* | -9.0 (8.5) | -12.9 (10.1) |

Data are presented as mean (SD).

*Calculated as 12 weeks minus baseline.

^a^ Of the 25 participants who did not complete the process measures at 12 weeks, 18 completed both primary outcomes and 7 did not. Results were calculated based on the 18 participants.
