# Supplementary material for: User Engagement and Experiences With an Online Unsupervised Tai Chi Program for People With Knee Osteoarthritis: Mixed Methods Process Evaluation Nested in a Randomized Controlled Trial
Source: JMIR Rehabil Assist Technol. 2025 Nov 14;12:e82115. doi: 10.2196/82115 (PMC12663698; doi:10.2196/82115)
Supplement: Multimedia Appendix 3 [file rehab_v12i1e82115_app3.docx]

**Multimedia Appendix 3**

**Fortnightly adherence rates in the Tai Chi group expressed as the percentage of participants classified as adherent (Tai Chi performed on average two or more days per week) and non-adherent, including those who did not provide adherence data categorized as non-adherent**


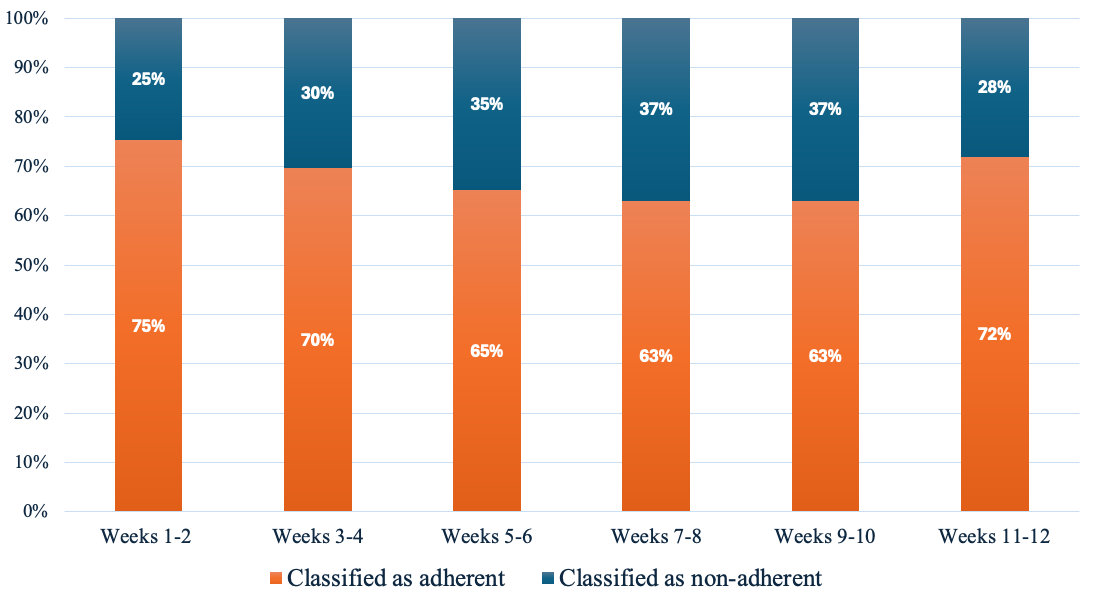


n=89 for each fortnightly period
